# Modifiable prognostic factors of high costs related to healthcare utilization among older people seeking primary care due to back pain: an identification and replication study

**DOI:** 10.1186/s12913-022-08180-2

**Published:** 2022-06-18

**Authors:** Rikke Munk Killingmo, Alessandro Chiarotto, Danielle A. van der Windt, Kjersti Storheim, Sita M. A. Bierma-Zeinstra, Milada C. Småstuen, Zinajda Zolic-Karlsson, Ørjan N. Vigdal, Bart W. Koes, Margreth Grotle

**Affiliations:** 1 Department of Physiotherapy, Oslo Metropolitan University, Oslo, Norway; 2 Department of General Practice, Erasmus MC, University Medical Centre, Rotterdam, The Netherlands; 3 School of Medicine, Keele University, Keele, UK; 4 Research and Communication Unit for Musculoskeletal Health (FORMI), Division of Clinical Neuroscience, Oslo University Hospital, Oslo, Norway; 5 Department of Orthopedics, Erasmus MC, University Medical Centre, Rotterdam, The Netherlands; 6 HTA and Reimbursement at Norwegian Medicines Agency, Oslo, Norway; 7 Center for Muscle and Joint Health, University of Southern Denmark, Odense, Denmark

**Keywords:** Back pain, Healthcare utilization, Costs, Prognostic factor research

## Abstract

**Background:**

Back pain is an extensive burden to our healthcare system, yet few studies have explored modifiable prognostic factors associated with high costs related to healthcare utilization, especially among older back pain patients. The aims of this study were to identify modifiable prognostic factors for high costs related to healthcare utilization among older people seeking primary care with a new episode of back pain; and to replicate the identified associations in a similar cohort, in a different country.

**Methods:**

Data from two cohort studies within the BACE consortium were used, including 452 and 675 people aged ≥55 years seeking primary care with a new episode of back pain. High costs were defined as costs in the top 25th percentile. Healthcare utilization was self-reported, aggregated for one-year of follow-up and included: primary care consultations, medications, examinations, hospitalization, rehabilitation stay and operations. Costs were estimated based on unit costs collected from national pricelists. Nine potential modifiable prognostic factors were selected based on previous literature. Univariable and multivariable binary logistic regression models were used to identify and replicate associations (crude and adjusted for selected covariates) between each modifiable prognostic factor and high costs related to healthcare utilization.

**Results:**

Four modifiable prognostic factors associated with high costs related to healthcare utilization were identified and replicated: a higher degree of pain severity, disability, depression, and a lower degree of physical health-related quality of life. Kinesiophobia and recovery expectations showed no prognostic value. There were inconsistent results across the two cohorts with regards to comorbidity, radiating pain below the knee and mental health-related quality of life.

**Conclusion:**

The factors identified in this study may be future targets for intervention with the potential to reduce high costs related to healthcare utilization among older back pain patients.

**Trial registration:**

ClinicalTrials.gov NCT04261309, 07 February 2020. Retrospectively registered.

**Supplementary Information:**

The online version contains supplementary material available at 10.1186/s12913-022-08180-2.

## Background

The burden of back pain has been growing along with an increasing and ageing population [1–5]. Back pain is the number one cause of disability globally [4] and an extensive burden to our healthcare systems [2, 6–8]. According to a recent systematic review, the prevalence rate of healthcare utilization for back pain ranges from 28 to 92% [9]. Back pain is one of the most prevalent complaints encountered in primary care [4, 5, 8, 10].

To improve use of scarce healthcare resources and reduce the burden on our healthcare systems, where possible and appropriate, researchers have highlighted the importance of monitoring and understanding healthcare utilization and related costs [3, 11]. It is well known that most of healthcare utilization and related costs stems from a relatively small group of back pain patients [12], and more importantly, that many of these patients receive unnecessary and ineffective treatment [3, 7]. This suggests that care for this high-cost subgroup requires quality improvement and cost reduction. An important next step towards this would be to identify modifiable prognostic factors associated with high costs related to healthcare utilization, and to replicate initial findings to evaluate the consistency of prognostic value across datasets and settings [13]. Information about such factors could inform development of effective strategies and/or interventions, or new applications of existing interventions.

Only a few prospective studies have explored modifiable prognostic factors associated with high costs related to healthcare utilization among patients with back pain [14–17], and no such study has been conducted among a sample of exclusively older people with back pain. Patients with high costs related to healthcare utilization are a diverse population [18, 19], and generalization of results cannot be done automatically from younger to older people with back pain [20]. With an ageing population, and the expected rise in older people requiring back care in the years to come [21], it is important to study modifiable prognostic factors of high costs related to healthcare utilization among older people with back pain.

Therefore, the aims of this study were 1) to identify modifiable prognostic factors for high costs related to healthcare utilization among older people seeking primary care with a new episode of back pain and 2) to replicate the identified associations in a similar cohort, in a different country.

## Method

This study was designed and performed in accordance with the PROGnosis RESearch Strategy (PROGRESS) framework [22], with aims consistent with prognostic factor research: identification of prognostic factors, including external replication. In line with recommendations from the PROGRESS framework [13] a study protocol (ClinicalTrials.gov NCT04261309, 07 February 2020) including a statistical analysis plan has been published [23], and the REMARK reporting guidelines were followed [24].

### Design and setting

This study was carried out in two steps. First, modifiable prognostic factors were identified in the Back Complaints in the Elderly - Norway study (BACE-N), a prospective observational cohort study within Norwegian primary care [25]. Next, a replication analysis was conducted in the Back Complaints in the Elders study (BACE-D), a prospective observational cohort study within Dutch primary care [26]. BACE-N has been classified as a quality assessment study by the Norwegian Regional Committee for medical Research Ethics (ref no. 2014/1634/REK vest) and approved by the Norwegian Social Science Data Service (ref no. 42149). Likewise, the BACE-D study protocol (NL24829.078.08) has been approved by the Medical Ethics Committee of the Erasmus Medical Center, the Netherlands. BACE-N and BACE-D are part of the international BACE consortium [26].

### Participants and recruitment procedure

Eligible participants within BACE-N were people ≥55 years of age seeking primary care (physiotherapist, chiropractor, or General Practitioner (GP)) with a new episode of back pain (preceded by 6 months without visiting primary care for similar complaints). Eligible participants within BACE-D were people > 55 years of age seeking primary care (GP) with a new episode of back pain (preceded by 6 months without visiting a GP for similar complaints). Patients were excluded from both studies if they had difficulties completing the questionnaires due to language barriers, or if they had difficulties completing the physical examination (e.g. are wheelchair bound). Patients within BACE-N were recruited by 110 physiotherapists, chiropractors and GPs in urban and rural parts of Norway between April 2015 and February 2020. Patients within BACE-D were recruited by 49 GPs in and around Rotterdam between March 2009 and September 2011. All included patients signed an informed consent form before study enrolment.

### Data collection, outcome, modifiable prognostic factors, and covariates

At baseline all patients responded to a comprehensive questionnaire and went through a standardized physical examination. Follow-up questionnaires were sent at 3, 6, and 12 months after inclusion within BACE-N and at 3, 6, 9 and 12 months after inclusion within BACE-D. All questionnaires were preferably completed electronically, but paper versions were available for patients not familiar with electronic data collection. Within this study, only data from questionnaires were used.

#### Outcome

The outcome of this study was costs related to healthcare utilization aggregated over one-year of follow up and dichotomized as high and low. Having high costs related to healthcare utilization was defined as patients with costs in the top 25th percentile [15, 16].

Healthcare utilization within BACE-N and BACE-D were self-reported and included: consultation to healthcare professionals (type and frequency), use of back medication (prescription and over-the-counter, type and frequency), number of diagnostic examinations (type and frequency), number of days of hospitalization and/or rehabilitation stay (within BACE-N) and back operations. Within BACE-N, consultations to healthcare professionals and use of back medication were reported with a 3-months recall period at each timepoint of follow-up. Number of diagnostic examinations and days of hospitalization and/or rehabilitation stay were reported with a 3-months recall period at 3- and 6-months follow-up, and a 6-months recall period at 12-months follow-up. Back operations were reported with a 12-months recall period at 12-months follow-up. Within BACE-D, all variables, except back operations, were reported with a 3-months recall period at each timepoint of follow-up. Back operations were reported with a 12-months recall period at 12-months follow-up. Total costs of healthcare utilization per patient were estimated by multiplying frequency of use by unit costs collected from national pricelists (see Table 1).

**Table 1:** Cost categories, units, unit price, all numbers in Euros (€) for 2020

Cost categories	Unit	Norwegian unit price (€)	Dutch unit price (€)	Reference (source)
*Primary care*				
General practitioner	Per visit	43.1	36.0	The Norwegian Medical Association, estimated average iMTA costing tool [27]
Occupational physician	Per visit	–	36.0	iMTA costing tool [27]
Physiotherapist	Per visit	47.2	36.0	The Norwegian Physiotherapy Association, estimated average iMTA costing tool [27]
Chiropractor	Per visit	55.0	36.0	Private price lists, estimated average. iMTA costing tool [27]
Manuel therapist	Per visit	74.2	36.0	The Norwegian Physiotherapy Association, estimated average iMTA costing tool [27]
Naprapath	Per visit	64.0	–	Private price lists, estimated average
Osteopath	Per visit	65.0	–	Private price lists, estimated average
Psychologist	Per visit	110.0	102.0	The Norwegian Psychological Association, estimated average iMTA costing tool [27]
Other therapists	Per visit	75.0	–	Private price lists, estimated average
*Back medication*				
Paracetamol	Per daily defined dose	0.5	0.9	NoMA price list, estimated average. Medicijnkosten.nl, estimated average incl. Pharmacy delivering costs [27]
NSAID	Per daily defined dose	1.2	0.4	NoMA price list, estimated average. Medicijnkosten.nl, estimated average incl. Pharmacy delivering costs [27]
Muscle relaxant	Per daily defined dose	0.7	0.5	NoMA price list, estimated average. Medicijnkosten.nl, estimated average incl. Pharmacy delivering costs [27]
Sleep medication	Per daily defined dose	0.2	–	NoMA price list, estimated average
Cortisone	Per daily defined dose	0.4	–	NoMA price list, estimated average
Opioid	Per daily defined dose	0.9	0.5	NoMA price list, estimated average. Medicijnkosten.nl, estimated average incl. Pharmacy delivering costs [27]
Antidepressant	Per daily defined dose	–	0.3	Medicijnkosten.nl, estimated average incl. Pharmacy delivering costs [27]
Anticonvulsant	Per daily defined dose	–	0.7	Medicijnkosten.nl, estimated average incl. Pharmacy delivering costs [27]
*Examinations*				
Blood sample	Per examination	20.4	4.4	The Norwegian Medical Association, estimated average iMTA costing tool [27]
X-ray	Per examination	119.0	45.9	Unilabs price list, estimated average The National Health Authority
MRI	Per examination	269.0	233.0	Unilabs price list, estimated average iMTA costing tool [27]
CT	Per examination	189.0	151.0	Unilabs price list, estimated average iMTA costing tool [27]
Secondary care				
Medical specialist	Per visit	–	125.0	iMTA costing tool [27]
Back operation	Per operation	5220.0	5254.0	DRG2150. Different academic and non-academic hospitals pricelists, estimated average
Hospitalization (non-operation)	Per day	1880.0	–	The Norwegian Directorate of Health, SAMDATA
Rehabilitation stay	Per day	315.0	–	UniCare pricelist, estimated average

*iMTA* indicates institute for Medical Technology Assessment, *NSAID* Non-steriodal anto-anflammatory drug, *NoMA* Norwegian Medicines Agency. Cells marked with a dash (−) indicate that the unit price was not estimated

#### Modifiable prognostic factors

Potential modifiable prognostic factors were factors expected to have the potential to be modified or improved by appropriate care or treatment, and therefore classified as modifiable. Potential modifiable prognostic factors of high costs related to healthcare utilization were based on previous scientific literature on (primarily) middle-aged back pain patients as well as patients with musculoskeletal disorders, and included the following self-reported variables measured at baseline:

- Pain severity [14–16, 28–30] measured by a Numeric Rating Scale (NRS) (range 0-10, higher score indicating higher pain severity) [31].
- Disability [14–16, 28, 29, 32] measured by the Roland-Morris Disability questionnaire (RMDQ) (range 0-24, higher score indicating higher degree of back-related disability) [33].
- Health-related quality of life [14, 30] measured by the Short-Form Health Survey 36-item (SF36) physical and mental summary score (range 0-100, higher score indicating better health-related quality of life) [34].
- Emotional well-being [15, 16, 19, 32, 35] measured by the Center for Epidemiological Studies-Depression questionnaire (CES-D) (range 0-60, higher score indicating more signs of depression) [36].
- Kinesiophobia [15, 35] measured by the Fear Avoidance Beliefs Questionnaire - Physical Activity subscale (FABQ-PA) (range 0-24, higher score indicating higher levels of kinesiophobia) [37].
- Comorbidity [17, 30] measured by the Self-Administered Comorbidity Questionnaire (SCQ) (range 0-15, thirteen pre-defined comorbidities and two optional comorbidities. Item no. 12 (back pain) was replaced with a third optional comorbidity) [38].
- Radiating pain below the knee [15] measured by the question “did your back pain radiate to your legs last week? If yes, how far down did the pain radiate?” and categorized into yes/no.
- Expectations of recovery from back pain within the next 3 months measured with a five-point scale and categorized into “recovered”, “much better” or “no change or worse”.

#### Covariates

Prognostic factor research may vary depending on context (time, place, healthcare setting) and characteristics of the study population. We therefore adjusted for potential covariates when evaluating the modifiable prognostic factors. Potential covariates were based on previous scientific literature on (primarily) middle-aged back pain patients as well as patients with musculoskeletal disorders, and included the following self-reported variables measured at baseline:

- Sex [14, 28, 39, 40] (female/male).
- Age [14, 28, 39, 40] (years).
- Education level [32, 41] measured as the highest education completed and categorised into low (elementary and high school level) or high (university level).
- Employment status measured by the question “do you have a paying job?” and categorized into yes/no.
- Pain duration [16] measured by the question “how many days have you had your current back pain?” and categorized into < 6 weeks, 6 weeks to 3 months or > 3 months.
- Pain history [29] measured by the question “have you had back pain before?” and categorized into yes/no.
- First healthcare provider [42] (physiotherapist, chiropractor, or GP).
- Total costs related to healthcare utilization during a period of 6 (BACE-N) or 12 (BACE-D) weeks prior to inclusion. Healthcare utilization prior to inclusion was self-reported and included: primary care consultations, use of back medication and number of diagnostic examinations. Total cost of healthcare utilization was estimated by multiplying frequency of use by unit costs collected from national pricelists (see Table 1).

### Analyses

All analyses are outlined in the statistical analysis plan published a priori [25] and preformed using the IBM SPSS version 26 (IBM Corporation, Armonk, NY, USA). We considered our study as explanatory. Thus, no correction for multiple testing was performed and *p*-values < 0.05 were considered statistically significant. All statistical tests were two-sided.

#### Study flow

The flow of patients through the studies were reported with a flow chart according to the REMARK guidelines [24]. Reasons for dropout were provided where known. Dropouts at 12-months follow-up were removed from the analyses. Differences in baseline characteristics between responders and non-responders at 12-months follow-up were evaluated.

#### Missing data

Whitin BACE-N, missing value pattern was visually explored, and missingness at random was assumed. Also, we found evidence against the hypothesis that values were not missing completely at random (Little’s test, *p* > 0.05). Missing baseline data was handled by multiple imputation. Five multiple imputation datasets with 10 iterations were created using regression estimation. We did not impute missing outcome values, as the imputation model had poor predictive performance and caused a clear trend of values being overestimated. Instead, missing values on variables used to estimate the outcome score were filled in with; 1) each patient’s individual average of observed values for the variables: consultations to healthcare professionals and medication use, 2) a value of zero costs for the variables: diagnostic examinations, hospitalization, rehabilitation stay and back operations. Within BACE-D, missing value pattern was visually explored, and missingness at random was assumed. Missing values on variables used to estimate the outcome score were filled in with; 1) each patient’s individual average of observed values for the variables: consultations to healthcare professionals and medication use, 2) a value of zero costs for the variables: diagnostic examinations and back operations.

#### Healthcare utilization and cost estimation

Type and frequency of use of different healthcare resources were calculated for each of the follow-up periods. Costs of healthcare utilization per patient were estimated by multiplying frequency of use by unit prices collected from national pricelists (see Table 1). Costs related to back medication were estimated based on medication type and frequency of use (data on dosage were not available). All costs were presented in Euros (€) for 2020 and estimated for the entire follow-up period with both mean and median values with 95% CI, using bias-corrected and accelerated (BCa) bootstrapping. The BCa was conducted with a bootstrap sample size of 1000. Cost data are commonly skewed thus both mean and median values were presented to inform interpretation. Norwegian prices were recalculated to Euros using the exchange rate from the National Bank of Norway from February 2020 (1€ = NOK 10).

#### Identification analysis

Univariable and multivariable binary logistic regression models were used to investigate associations (crude and adjusted for selected covariates) between each predefined modifiable prognostic factor and costs related to healthcare utilization (within BACE-N). The cost score was entered into the model as a dependent dichotomous variable (high costs defined as patients with cost in the top 25th percentile, yes/no). Linearity of continuous independent variables were examined using Box-Tidwell transformations [43]. Independent variables that demonstrated a non-linear relationship with the dependent variable where categorized. The results were presented as crude and adjusted odds ratios (OR) with 95% CI.

#### Replication analysis

Univariable and multivariable binary logistic regression models were used, as described above, to replicate findings from the identification analysis within BACE-D. The results were presented as crude and adjusted OR with 95% CI. The decision on whether findings were replicated were based on the direction and magnitude of the association, and the size of the CI for each of the predefined modifiable prognostic factors [44].

#### Sensitivity analysis

To assess credibility of the identification analysis and possible bias introduced by the imputation procedure, the univariable and multivariable logistic regression analyses were performed on complete case data (within BACE-N).

#### Sample size

This study contains secondary analyses embedded in the BACE-N and BACE-D. Details of the sample size calculation related to the original aims of the cohorts are provided in the BACE-N and BACE-D protocols [25, 26]. To determine statistical power for this particular study, we used number of events per variable (EPV) [45–49] and the rule-of-thumb of “10 events per 1 analysed variable” [50–53]. With a sample size of 450 participants within BACE-N, we anticipated 112 participants to be in the top 25th percentile of costs and categorized as having high costs (yes/no) (events). An EPV of 10 would allow a maximum of 11 prognostic variables to be included in the final multivariable prediction model. With a sample size of 675 participants in BACE-D, we anticipated 168 participants to be in the top 25th percentile of costs and defined as having high costs (yes/no) (events). An EPV of 10 would allow a maximum of 16 prognostic variables to be included in the final multiple prediction model.

## Results

**Fig. 1:**
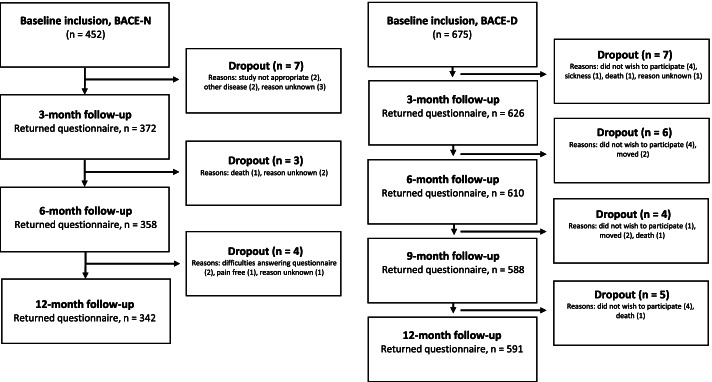
Participant flow chart BACE-N and BACE-D

A total of 452 (BACE-N) and 675 (BACE-D) patients were included in the identification and the replication sample, respectively. Table 2 shows patient characteristics and clinical status at baseline, along with the proportion of missing data per variable. Flow of patients through the studies are shown in Fig. 1. Fourteen patients (3%) in BACE-N and 22 patients (3%) in BACE-D were dropouts at 12-months follow-up. We removed these cases from the analyses. Within BACE-N, there was a larger proportion of females (55 vs. 42%) among the responders as compared to non-responders. Within BACE-D, there was a larger proportion of people not in paid work (26 vs. 38%) and people with short pain duration < 6 weeks (56 vs. 39%) among the responders as compared to non-responders. Otherwise, there were no differences between responders and non-responders in the two cohorts. The BACE-N and BACE-D samples were also largely comparable, although there were some differences that might have impacted healthcare utilization. BACE-N had a larger proportion of people with high education level (44 vs. 17%), people in paid work (47 vs. 27%), and people with short pain duration < 6 weeks (67 vs. 54%).

**Table 2:** Patient characteristics and clinical status at baseline in the identification and replication sample*

	BACE-N		BACE-D	
	All participants (*n =* 452)	Missing, n (%)	All participants (*n =* 675)	Missing, n (%)
Female	235 (52)	0 (0)	401 (59)	0 (0)
Age in years	66 (59-72)	0 (0)	65 (60-71)	0 (0)
Education level high	188 (44)	20 (4)	114 (17)	7 (1)
Ethnicity Norwegian (BACE-N) or Dutch (BACE-D)	430 (95)	0 (0)	637 (96)	10 (1)
Employment status currently paid work	211 (47)	5 (1)	177 (27)	23 (3)
First healthcare provider				
General practitioner	127 (28)	0 (0)	675 (100)	0 (0)
Physiotherapist	130 (29)	0 (0)	0 (0)	0 (0)
Chiropractor	195 (43)	0 (0)	0 (0)	0 (0)
Pain location				
Thoracic	56 (13)	11 (2)	154 (26)	71 (11)
Lumbar/Sacral	406 (92)	11 (2)	561 (93)	71 (11)
Radiating pain below the knee	141 (31)	0 (0)	205 (31)	7 (1)
Pain severity average last week (NRS, 0-10)	5 (4-7)	31 (7)	5 (3-7)	11 (2)
Pain duration				
< 6 weeks	252 (67)	76 (17)	323 (54)	80 (12)
6 weeks to 3 months	49 (13)	76 (17)	116 (20)	80 (12)
> 3 months	75 (20)	76 (17)	156 (26)	80 (12)
Previous episodes of back pain	400 (95)	29 (6)	579 (86)	9 (1)
Disability (RMDQ, 0-24)	9 (4-13)	45 (10)	10 (5-14)	55 (8)
Comorbidity (SCQ, 0-15)	1 (1-2)	18 (4)	2 (1-3)	6 (1)
Health-related QOL (SF36, 0-100)				
Physical component	42 (36-47)	41 (9)	43 (37-50)	7 (1)
Mental component	55 (48-60)	41 (9)	52 (43-57)	7 (1)
Emotional well-being (CES-D, 0-60)	8 (3-13)	57 (13)	9 (4-14)	57 (8)
Kinesiophobia (FABQ-PA, 0-24)	9 (5-13)	18 (4)	14 (10-17)	20 (3)
Expectations of recovery within 3 months				
Fully recovered	111 (26)	19 (4)	113 (17)	17 (2)
Much better	217 (50)	19 (4)	178 (27)	17 (2)
No change or worse	105 (24)	19 (4)	367 (56)	17 (2)
*Healthcare utilization prior to inclusion*				
Primary care consultation last 6 (BACE-N) or 12 (BACE-D) weeks				
General practitioner	78 (18)	21 (5)	609 (91)	8 (1)
Occupational physician	–	–	13 (2)	8 (1)
Physiotherapist, Chiropractor or Manual therapist	295 (68)	21 (5)	299 (45)	8 (1)
Psychologist	2 (0.5)	21 (5)	5 (1)	8 (1)
Other therapists	21 (5)	21 (5)	–	–
Use of back medication	165 (40)	38 (8)	484 (73)	8 (1)
Diagnostic examination last 6 (BACE-N) or 3 (BACE-D) months				
Blood sample	12 (3)	24 (5)	92 (14)	10 (2)
X-ray	23 (5)	24 (5)	155 (23)	10 (2)
MRI/CT scan	49 (11)	24 (5)	30 (5)	10 (2)
Previous hospitalization	48 (11)	21 (5)	–	–
Previous rehabilitation stay	17 (4)	25 (6)	–	–
Medical specialist consultation	–	–	46 (7)	8 (1)

*CES-D* indicates The Center for Epidemiologic Studies Depression Scale, *FABQ-PA* The Fear Avoidance Beliefs Questionnaire, physical activity subscale, *NRS* Numeric Rating Scale, *RMDQ* The Roland Morris Disability Questionnaire, *SCQ* The Self-Administered Comorbidity Questionnaire, *SF-36* The Short-Form Health Survey 36-item. *The presented characteristics are based on complete case data. All values are presented by number (valid percentage of total) or median (IQR). Cells marked with a dash (−) indicate that the variable was not measured

**Table 3:** Costs (€) due to healthcare utilization from 0 to 12 month in the identification and replication sample*

	BACE-N (*n =* 438)			BACE-D (*n =* 653)		
	Mean (95% CI**)	Median (95% CI**)	Patients with zero cost, n (%)	Mean (95% CI**)	Median (95% CI**)	Patients with zero cost, n (%)
Primary care	458 (404-516)	242 (192-330)	83 (21)	289 (255-329)	72 (72-72)	250 (40)
Medication	52 (43-61)	3 (1-7)	176 (44)	62 (54-70)	7 (0-17)	291 (46)
Examination	65 (50-81)	0 (0-0)	308 (77)	73 (63-87)	0 (0-4)	342 (54)
Secondary care	243 (116-388)	0 (0-0)	390 (97)	158 (110-213)	0 (0-0)	503 (80)
**Total**	825 (682-976)	364 (307-440)	52 (13)	582 (506-666)	233 (190-276)	136 (22)

*Costs due to healthcare utilization for the entire follow-up period is calculated on basis of the three (BACE-N) and four (BACE-D) follow-up periods. Costs in the two cohorts are not direct comparable. The BACE-N lack data on primary care consultations and medication use between 6 and 9 months. Thus, total costs within the BACE-N are expected to be slightly underestimated. **Bias-corrected and accelerated bootstrapping (1000 simulations)

Within BACE-N, missing data ranged from 0.0 to 16.8% for included baseline variables and 18.4 to 26.0% for included follow-up variables. Total missingness was 4.9 and 23.3% for all baseline and follow-up values, respectively. Variables on medication use at 12-months follow-up had most missing values. Within BACE-D, missing data ranged from 0.0 to 11.8% for included baseline variables and 7.3 to 18.1% for included follow-up variables. Total missingness was 2.2 and 9.6% for all baseline and follow-up values, respectively. Variables on examination at 12-months follow-up had most missing values.

### Healthcare utilization and cost estimation

Table A1 and A2 in the Additional file 1 and Additional file 2 shows healthcare utilization throughout one-year of follow-up for the BACE-N and BACE-D sample, respectively. Costs related to healthcare utilization aggregated for the one-year of follow-up are shown in Table 3. Within BACE-N, 87% of all patients used healthcare during the one-year of follow-up, and a total of 110 patients (25%) were defined as having high costs (≥ € 789). Within BACE-D, 78% of all patients used healthcare during the one-year of follow-up, and a total of 163 patients (25%) were defined as having high costs (≥ € 664).

### Identification analysis

All continuous independent variables, aside from the two SF-36 variables, demonstrated a linear relationship with the dependent variable. Table 4 shows crude and adjusted OR with 95% CI for the association between each of the modifiable prognostic factor and being in the high costs group. All analyses showed a statistical significant crude association between the factors and the outcome. After adjustment for covariates, only the following factors remained significantly associated with the outcome: pain severity, disability, depression, comorbidity, radiating pain below the knee, and physical and mental health-related quality-of-life.

**Table 4:** Binary logistic regression analyses; individual associations between modifiable prognostic factors and high costs

	BACE-N (*n =* 438)		BACE-D (*n =* 653)	
	Crude OR (95% CI)	Adjusted OR* (95% CI)	Crude OR (95% CI)	Adjusted OR* (95% CI)
Pain severity (NRS, 0-10)	1169 (1.059-1.291)	1.147 (1.031-1.277)	1.295 (1.198-1.400)	1.324 (1.203-1.457)
Disability (RMDQ, 0-24)	1146 (1.096-1.198)	1.140 (1.087-1.195)	1.143 (1.101-1.186)	1.143 (1.092-1.196)
Emotional well-being (CES-D, 0-60)	1.050 (1.024-1.076)	1.040 (1.013-1.068)	1.041 (1.017-1.065)	1.038 (1.010-1.066)
Kinesiophobia (FABQ-PA, 0-24)	1.050 (1.012-1.090)	1.030 (0.990-1.071)	1.037 (1.005-1.071)	1.034 (0.997-1.073)
Comorbidity (SCQ, 0-15)	1.611 (1.363-1.905)	1.614 (1.339-1.945)	1.179 (1.051-1.323)	1.091 (0.948-1.254)
Radiating pain below knee (ref: no)	2.604 (1.662-4.080)	2.254 (1.389-3.660)	2.124 (1.468-3.073)	1.507 (0.969-2.345)
Health-related QOL physical (SF36, 0-100) (ref. 4. percentile)				
3. percentile	2.731 (1.237-6.029)	2.167 (0.956-4.909)	2.876 (1.649-5.016)	2.328 (1.222-4.437)
2. percentile	3.836 (1.774-8.296)	2.778 (1.250-6.173)	2.406 (1.440-4.021)	2.198 (1.221-3.958)
1. percentile	7.185 (3.377-15.290)	4.913 (2.235-10.803)	4.326 (2.562-7.303)	3.937 (2.082-7.445)
Health-related QOL mental (SF36, 0-100) (ref. 4. percentile)				
3. percentile	0.917 (0.452-1.859)	1.095 (0.523-2.292)	0.861 (0.473-1.556)	0.813 (0.410-1.613)
2. percentile	2.092 (1.104-3.961)	2.162 (1.102-4.240)	0.503 (0.272-0.933)	0.382 (0.187-0.784)
1. percentile	2.717 (1.444-5.113)	2.583 (1.317-5.068)	1.375 (0.779-2.367)	1.173 (0.629-2.185)
Expectations of recovery within 3 months (ref. recovered)				
Much better	2.321 (1.300-4.144)	1.622 (0.878-2.997)	1.552 (0.849-2.840)	1.129 (0.583-2.186)
No change or worse	1.547 (0.784-3.053)	1.004 (0.483-2.087)	1.887 (1.093-3.257)	1.275 (0.680-2.391)

*CES-D* indicates The Center for Epidemiologic Studies Depression Scale, *CI* Confidence interval, *FABQ-PA* The Fear Avoidance Beliefs Questionnaire, physical activity subscale, *NRS* Numeric Rating Scale, *OR* Odds ratio, *RMDQ* The Roland Morris Disability Questionnaire, *SCQ* The Self-Administered Comorbidity Questionnaire, *SF-36* The Short-Form Health Survey 36-item. *Adjusted by sex, age, education level, employment status, pain duration, pain history, first healthcare provider and costs related to healthcare utilization prior to inclusion

The sensitivity analysis (Table A3 in the Additional file 3) showed no substantial change in point estimates when comparing complete case analysis to the main analysis. There were some minor changes in *p*-values for the two SF-36 variables: In the complete case analysis of crude associations, the SF-36 mental second percentile group were not significantly associated with the outcome, and in the complete case analysis of adjusted associations, the SF-36 physical and mental second percentile groups were not significantly associated with the outcome.

### Replication analysis

Table 4 also shows results of the replication analysis. Except for the SF-36 mental second percentile group, findings were replicated with respect to the direction of the association between each of the factors and the outcome. Though, the magnitude of the association varied > 20% for the following factors: comorbidity, radiating pain below the knee, and physical and mental health-related quality-of-life.

In both the identification and replication analysis, after adjustment for selected covariates and with the “low cost group” as the reference, factors associated with increased odds of being in the high costs group were a higher degree of pain severity, disability and depression, and a lower degree of physical health-related quality of life. No association was found between being in the high costs group and the degree of kinesiophobia or expectations of recovery.

## Discussion

The present study identified and replicated associations between modifiable prognostic factors and high costs related to healthcare utilization among older people seeking primary care with a new episode of back pain. Four modifiable prognostic factors associated with high costs related to healthcare utilization were identified and replicated in a similar cohort, in a different country, reflecting slightly different sociodemographic characteristics and healthcare setting: pain severity, disability, depression and physical health-related quality of life. Kinesiophobia and expectations of recovery showed no prognostic value. There were inconsistent results across the two cohorts with regards to comorbidity, radiating pain below the knee and mental health-related quality of life.

To the best of our knowledge, no similar study has been conducted among a sample of exclusively older people with back pain. Thus, direct comparability of this study with other studies is limited. Nevertheless, our findings are generally in accordance with previous research on (primarily) middle-aged back pain patients [14–16, 28, 29, 32, 35], as well as patients with musculoskeletal disorders [30, 54, 55]. For example, pain severity, disability and depression have been shown to be significantly associated with high costs related to healthcare utilization in studies on patients with back pain [14–16, 28, 29, 32, 35] and musculoskeletal disorders [30, 54, 55]. Physical health-related quality of life has also previously been reported to be a prognostic factor of high societal costs among back pain patients [14], and high costs related to healthcare utilization among patients with musculoskeletal disorders [30]. Our findings regarding kinesiophobia and radiating pain below the knee are also in line with a previous study [15], which showed that these factors were of minor importance when predicting future costs related to healthcare utilization among back pain patients. Our finding regarding mental health-related quality of life is, however, contrary to a study on patients with musculoskeletal disorders [30], which found that this factor was associated with persistent high costs related to healthcare utilization. Our finding regarding comorbidity is also contrary to previous research. In a recent systematic review, comorbidity was pointed out as a consistent prognostic factor of high costs related to healthcare utilization in general [18], and similar conclusions have been drawn in single studies among patients with back pain [17] and musculoskeletal disorders [55]. This discrepancy might be explained by the fact that we included costs related to back pain specific healthcare utilization, whereas other studies have included healthcare costs related to all musculoskeletal disorders [17, 55] and healthcare costs in general [18]. To the best of our knowledge, the prognostic value of recovery expectations for high costs related to healthcare utilization has not been reported previously.

The main limitation of this study is missing data on variables used to estimate the outcome score, thus we had to manually replace missing values. It is well-known that healthcare utilization is prone to missing data [56–58]. Also, that missing data should be replaced in order to make use of all reported data [56, 57]. Unfortunately, due to poor predictive performance, multiple imputation could not be used on follow-up data in this study. We therefore chose a frequently used, though not optimal, method for replacing missing values [58] and have tried to be transparent in our reporting. A second potential limitation is that we used self-reported data on healthcare utilization. Self-reports tend to underestimate the true value of healthcare utilization due to potential recall bias [59–62]. Nevertheless, we consider the impact of recall bias to be of only minor importance in this study as the outcome variable was dichotomized into high or low costs. In future studies, the limitations of missing data and recall bias could to some extent be overcome by including registry data on healthcare utilization. A third potential limitation is that costs related to hospitalization and rehabilitation stays were not measured in BACE-D. Thus, the risk of misclassification bias related to whether patients were classified as having high or low costs might be present in BACE-D. However, if costs related to hospitalization and rehabilitation stays were removed from the cost calculations in BACE-N, only 4 patients (< 1%) switched cost group. A fourth potential limitation is that we could not adjust for possibly important covariates of healthcare utilization, such as the patient’s disposition to access and pay for healthcare services, and health insurance status. According to the Behavioral Model of Health Services Use from Andersen [63], healthcare utilization is a function of people’s predisposition to use services, factors which enable or impede use and need for care. Certainly, including these enabling factors is recommended. However, it is likely to assume that these factors are of less importance in countries such as Norway and the Netherlands, where health services are largely available and covered by the public sector. A fifth limitation is the lack of data on eligible participants that declined to participate or for other reasons were not invited. Due to limited resources and practical reasons related to recruitment from a broad network of clinicians, it was not possible to record information on all eligible participants during the BACE-N and BACE-D data collection period. Thus, the risk of selection bias is present. To compensate for this limitation and assess representativeness of the BACE-N sample, key sociodemographic variables have been compared with a large population study on older people; The Norwegian study on life course, ageing and generation (NORLAG) [64, 65]. A subsample of the NORLAG (NORLAG MSK) was used, which is expected to be a representative sample of people aged ≥55 years with musculoskeletal complaints. Characteristics of the two samples were largely comparable, though BACE-N had more men, and more with higher education level [65]. Previous studies have shown that women [14, 28, 29] are more likely to seek care for their back pain as are people with lower education level [28, 32, 41]. Hence, it is likely to assume that the amount of healthcare utilization presented in BACE-N is somewhat underestimated. Furthermore, the BACE-N sample is largely comparable to younger Norwegian back pain cohorts [66, 67] and to the BACE-D sample [26].

The main strength of the present study is that it was conducted in line with the PROGRESS framework including an identification and replication phase [13], pre-planned with a published statistical analysis plan, and reported in line with the REMARK guidelines [24]. Also, that it estimates the prognostic value of modifiable prognostic factors over and beyond a core set of non-modifiable covariates. Prognostic factor studies are an essential step towards quality improvement of clinical practice [13]. Results from such studies have the potential to inform development of effective strategies and/or interventions. Identifying modifiable prognostic factors of high costs related to healthcare utilization among older people is an important step towards addressing the global burden of back pain and decrease waste of valuable healthcare resources [3, 7, 68].

## Conclusion

In conclusion, this study identified and replicated four modifiable prognostic factors associated with high costs related to healthcare utilization among older people seeking primary care with a new episode of back pain: pain severity, disability, depression, and physical health-related quality of life. This study contributes to the on-going research into clinical pathways and has the potential to identify future target areas for intervention with the potential to reduce high costs related to healthcare utilization among older back pain patients. Due to differences in healthcare systems between countries, readers are advised to exercise caution with generalizability of the results to other healthcare systems.

## Supplementary Information

**Additional file 1.**

**Additional file 2.**

**Additional file 3.**

## Data Availability

Data supporting findings of this study are not public available as participants have consented for their data to be available only to the researcher of this study. However, data are available from the corresponding author upon reasonable request and with permission of the Oslo Metropolitan University and the Erasmus Medical Center (contact through the corresponding author).

## Abbreviations

BCa: Bias-corrected and accelerated
BACE-D: Back Complaints in the Elders study
BACE-N: Back Complaints in the Elderly - Norway study
CES-D: Center for Epidemiological Studies-Depression questionnaire
CI: Confidence Interval
EPV: Events per variable
FABQ-PA: Fear Avoidance Beliefs Questionnaire - Physical Activity subscale
GP: General Practitioner
NOK: Norwegian krone
NORLAG: Norwegian study on life course, ageing and generation
NRS: Numeric Rating Scale
OR: Odds ratio
PROGRESS: PROGnosis RESearch Strategy
RMDQ: Roland-Morris Disability Questionnaire
REMARK: REporting recommendations for tumor MARKer prognostic studies
SCQ: Self-Administered Comorbidity Questionnaire
SF36: Short-Form Health Survey 36-item
