# Supplementary material for: Modifiable prognostic factors of high costs related to healthcare utilization among older people seeking primary care due to back pain: an identification and replication study
Source: BMC Health Serv Res. 2022 Jun 18;22:793. doi: 10.1186/s12913-022-08180-2 (PMC9206382; doi:10.1186/s12913-022-08180-2)
Supplement: Supplementary file 1 — Additional file 1. [file 12913_2022_8180_MOESM1_ESM.docx]

**Additional file 1:**

| **Table A1.** Healthcare utilization throughout one-year of follow-up BACE-N (n=438) | | | | | | |
| --- | --- | --- | --- | --- | --- | --- |
|  | 0-3 months | | >3-6 months | | >9-12 months | |
|  |  | Missing,  n (%) |  | Missing,  n (%) |  | Missing, n (%) |
| *Primary care* |  |  |  |  |  |  |
| Patients with primary care consultation, n (%) |  | 79 (18) |  | 87 (20) |  | 108 (24) |
| GP | 44 (12) |  | 30 (9) |  | 22 (7) |  |
| Physiotherapist | 119 (33) |  | 70 (20) |  | 48 (15) |  |
| Chiropractor | 124 (35) |  | 76 (22) |  | 50 (15) |  |
| Manual therapist | 22 (6) |  | 5 (1) |  | 7 (2) |  |
| Naprapath | 6 (2) |  | 11 (3) |  | 6 (2) |  |
| Osteopath | 2 (0.6) |  | 1 (0.3) |  | 3 (1) |  |
| Psychologist | 0 (0) |  | 1 (0.3) |  | 1 (0.3) |  |
| Other therapists | 10 (3) |  | 12 (3) |  | 7 (2) |  |
| No primary care consultations | 93 (26) |  | 179 (51) |  | 212 (64) |  |
| Numbers of consultations, median (IQR)* |  | 0 (0) |  |  |  |  |
| GP | 1 (1-2) |  | 1 (1-2) | 0 (0) | 1 (1-3) | 0 (0) |
| Physiotherapist | 4 (2-8) |  | 4 (2-10) | 2 (3) | 5 (1-9) | 0 (0) |
| Chiropractor | 4 (2-6) |  | 2 (1-4) | 4 (5) | 3 (1-5) | 0 (0) |
| Manual therapist | 3 (1-5) |  | 3 (2-14) | 0 (0) | 1 (1-4) | 0 (0) |
| Naprapath | 3 (1-5) |  | 4 (2-6) | 0 (0) | 3 (1-4) | 0 (0) |
| Osteopath | 3 (2-) |  | 2 (2-2) | 0 (0) | 10 (2-) | 0 (0) |
| Psychologist | - |  | 1 (1-1) | 0 (0) | 7 (7-7) | 0 (0) |
| Other consultations | 4 (1-6) |  | 1 (1-8) | 0 (0) | 4 (2-8) | 1 (14) |
| *Back medication* |  |  |  |  |  |  |
| Patients with use of back medication, n (%) |  | 80 (18) |  | 96 (22) |  | 114 (26) |
| Paracetamol | 124 (35) |  | 91 (27) |  | 86 (27) |  |
| NSAID | 86 (24) |  | 75 (22) |  | 64 (20) |  |
| Muscle relaxants | 6 (2) |  | 4 (1) |  | 3 (1) |  |
| Sleep medication | 22 (6) |  | 22 (6) |  | 13 (4) |  |
| Cortisone | 5 (1) |  | 9 (3) |  | 4 (1) |  |
| Opioid | 5 (1) |  | 5 (2) |  | 3 (1) |  |
| No use of back medication | 197 (55) |  | 213 (62) |  | 213 (66) |  |
| Frequency of use paracetamol, n (%)** |  | 0 (0) |  | 0 (0) |  | 0 (0) |
| Daily | 46 (37) |  | 32 (35) |  | 30 (35) |  |
| Weekly | 35 (28) |  | 30 (33) |  | 28 (33) |  |
| Monthly or less | 43 (35) |  | 29 (32) |  | 28 (32) |  |
| Frequency of use NSAID, n (%)** |  | 0 (0) |  | 0 (0) |  | 0 (0) |
| Daily | 22 (26) |  | 16 (21) |  | 17 (26) |  |
| Weekly | 14 (16) |  | 25 (33) |  | 19 (30) |  |
| Monthly or less | 50 (58) |  | 34 (46) |  | 28 (44) |  |
| Frequency of use sleep opioid, n (%)** |  | 0 (0) |  | 0 (0) |  | 0 (0) |
| Daily | 3 (60) |  | 4 (80) |  | 2 (67) |  |
| Weekly | 1 (20) |  | - |  | - |  |
| Monthly or less | 1 (20) |  | 1 (20) |  | 1 (33) |  |
| *Examinations* |  |  |  |  |  |  |
| Patients with diagnostic examination, n (%) |  | 79 (18) |  | 86 (20) |  | 106 (24) |
| Blood sample | 9 (3) |  | 5 (1) |  | 6 (2) |  |
| X-ray | 12 (3) |  | 8 (2) |  | 16 (5) |  |
| MRI | 37 (10) |  | 17 (5) |  | 20 (6) |  |
| CT | 4 (1) |  | 2 (1) |  | 2 (1) |  |
| No diagnostic examination | 281 (77) |  | 316 (89) |  | 289 (87) |  |
| *Secondary care* |  |  |  |  |  |  |
| Back operation, n (%) | - |  | - |  | 7 (2) | 103 (24) |
| Patients with hospitalization, n (%) | 5 (1) | 75 (17) | 6 (2) | 84 (19) | 2 (1) | 104 (24) |
| Duration of stay in days, median (range) | 1 (1-2) | 0 (0) | 3 (2-5) | 1 (17) | 2.5 (2-) | 0 (0) |
| Patients with rehabilitation stay, n (%) | 0 (0) | 73 (17) | 1 (0.3) | 84 (19) | 1 (0.3) | 104 (24) |
| Duration of stay in days, median (range) | - |  | 20 (20-20) | 0 (0) | 7 (7-7) | 0 (0) |
| NSAID indicates non-steriodal anto-anflammatory drug. *Numbers of consultations is calculated on basis of patients who have reported primary care consultations. **Frequency of back medication use is calculated on basis of patients who have reported back medication use. Cells marked with a dash (-) indicate that the variable was not reported. | | | | | | |
