# Supplementary material for: Modifiable prognostic factors of high costs related to healthcare utilization among older people seeking primary care due to back pain: an identification and replication study
Source: BMC Health Serv Res. 2022 Jun 18;22:793. doi: 10.1186/s12913-022-08180-2 (PMC9206382; doi:10.1186/s12913-022-08180-2)
Supplement: Supplementary file 2 — Additional file 2. [file 12913_2022_8180_MOESM2_ESM.docx]

**Additional file 2:**

| **Table A2.** Healthcare utilization throughout one-year of follow-up BACE-D (n=653) | | | | | | | | |
| --- | --- | --- | --- | --- | --- | --- | --- | --- |
|  | 0-3 months | | >3-6 months | | >6-9 months | | >9-12 months | |
|  |  | Missing,  n (%) |  | Missing,  n (%) |  | Missing,  n (%) |  | Missing,  n (%) |
| *Primary care* |  |  |  |  |  |  |  |  |
| Primary care consultation, n (%) |  | 46 (7) |  | 58 (9) |  | 92 (14) |  | 86 (13) |
| General practitioner | 158 (26) |  | 75 (13) |  | 68 (12) |  | 57 (10) |  |
| Physiotherapist, Chiropractor or Manual  therapist | 217 (36) |  | 145 (24) |  | 104 (19) |  | 103 (18) |  |
| Psychologist | 3 (0.5) |  | 3 (0.5) |  | 2 (0.4) |  | 4 (1) |  |
| Occupational physician | 15 (3) |  | 8 (1) |  | 7 (1) |  | 4 (1) |  |
| No primary care consultations | 294 (48) |  | 183 (31) |  | 142 (25) |  | 139 (25) |  |
| Numbers of consultations, median (IQR)* |  |  |  |  |  |  |  |  |
| General practitioner | 1 (1-2) | 4 (3) | 1 (1-2) | 3 (4) | 1 (1-2) | 2 (3) | 1 (1-2) | 2 (4) |
| Physiotherapist | 6 (3-10) | 5 (2) | 5 (3-10) | 5 (3) | 5 (2-10) | 2 (2) | 5 (2-10) | 1 (1) |
| Psychologist | 3 (1-) | 0 (0) | 2 (2-) | 0 (0) | 3 (1-) | 0 (0) | 2 (1-3) | 0 (0) |
| Occupational physician | 1 (1-3) | 0 (0) | 2 (1-3) | 0 (0) | 1 (1-3) | 0 (0) | 2 (1-3) | 0 (0) |
| *Back medication* |  |  |  |  |  |  |  |  |
| Use of back medication, n (%) |  | 46 (7) |  | 60 (9) |  | 95 (15) |  | 86 (13) |
| Paracetamol | 118 (19) |  | 109 (18) |  | 118 (21) |  | 109 (19) |  |
| NSAID | 108 (17) |  | 94 (16) |  | 81 (15) |  | 64 (11) |  |
| Opioid | 49 (8) |  | 35 (6) |  | 31 (6) |  | 38 (7) |  |
| Muscle relaxants or sleep medication | 9 (2) |  | 6 (1) |  | 6 (1) |  | 5 (1) |  |
| Antidepressant | 7 (1) |  | 5 (1) |  | 3 (1) |  | 2 (0.4) |  |
| Anticonvulsant | 4 (1) |  | 4 (1) |  | 6 (1) |  | 7 (1) |  |
| No use of back medication | 360 (59) |  | 387 (65) |  | 359 (64) |  | 380 (67) |  |
| Frequency of use paracetamol, n (%)** |  | 6 (5) |  | 3 (3) |  | 7 (6) |  | 6 (5) |
| Daily | 45 (40) |  | 38 (36) |  | 39 (35) |  | 41 (40) |  |
| Weekly | 60 (54) |  | 58 (55) |  | 61 (55) |  | 54 (52) |  |
| Less than weekly | 7 (6) |  | 10 (9) |  | 11 (10) |  | 8 (8) |  |
| Frequency of use NSAID, n (%)** |  | 1 (1) |  | 2 (2) |  | 2 (3) |  | 0 (0) |
| Daily | 50 (47) |  | 36 (39) |  | 27 (34) |  | 26 (41) |  |
| Weekly | 40 (37) |  | 42 (46) |  | 40 (51) |  | 29 (45) |  |
| Less than weekly | 17 (16) |  | 14 (15) |  | 12 (15) |  | 9 (14) |  |
| Frequency of use sleep opioid, n (%)** |  | 1 (2) |  | 2 (6) |  | 1 (3) |  | 1 (3) |
| Daily | 32 (67) |  | 23 (70) |  | 22 (73) |  | 27 (73) |  |
| Weekly | 14 (29) |  | 8 (24) |  | 6 (20) |  | 8 (22) |  |
| Less than weekly | 2 (4) |  | 2 (6) |  | 2 (7) |  | 2 (5) |  |
| *Examinations* |  |  |  |  |  |  |  |  |
| Diagnostic examination, n (%) |  | 55 (8) |  | 65 (10) |  | 101 (16) |  | 88 (14) |
| Blood sample | 82 (14) |  | 46 (8) |  | 34 (6) |  | 26 (5) |  |
| X-ray | 181 (30) |  | 57 (10) |  | 39 (7) |  | 30 (5) |  |
| MRI/CT | 41 (7) |  | 41 (7) |  | 25 (5) |  | 27 (5) |  |
| No diagnostic examination | 210 (35) |  | 88 (15) |  | 63 (11) |  | 55 (10) |  |
| *Secondary care* |  |  |  |  |  |  |  |  |
| Medical specialist consultation, n (%) | 66 (11) | 46 (7) | 55 (9) | 58 (9) | 49 (9) | 92 (14) | 45 (8) | 86 (13) |
| Back operation, n (%) | - |  | - |  | - |  | 10 (2) | 94 (14) |
| NSAID indicates non-steriodal anto-anflammatory drug. *Numbers of consultations is calculated on basis of patients who have reported primary care consultations. **Frequency of back medication use is calculated on basis of patients who have reported back medication use. Cells marked with a dash (-) indicate that the variable was not reported. | | | | | | | | |
