# Supplementary material for: Modifiable prognostic factors of high costs related to healthcare utilization among older people seeking primary care due to back pain: an identification and replication study
Source: BMC Health Serv Res. 2022 Jun 18;22:793. doi: 10.1186/s12913-022-08180-2 (PMC9206382; doi:10.1186/s12913-022-08180-2)
Supplement: Supplementary file 3 — Additional file 3. [file 12913_2022_8180_MOESM3_ESM.docx]

**Additional file 3:**

| **Table A3.** Sensitivity Binary logistic regression analyses; individual associations between modifiable prognostic factors and high costs (BACE-N) | | | | |
| --- | --- | --- | --- | --- |
|  | Crude OR (95% CI) | Missing, n (%) | Adjusted OR* (95% CI) | Missing, n (%) |
| Pain severity (NRS, 0-10) | 1.16 (1.05-1.28) | 29 (7) | 1.17 (1.02-1.33) | 126 (29) |
| Disability (RMDQ, 0-24) | 1.14 (1.09-1.19) | 43 (10) | 1.15 (1.09-1.22) | 143 (33) |
| Emotional well-being (CES-D, 0-60) | 1.07 (1.04-1.10) | 51 (12) | 1.07 (1.03-1.11) | 147 (34) |
| Kinesiophobia (FABQ-PA, 0-24) | 1.05 (1.01-1.09) | 16 (4) | 1.02 (0.98-1.07) | 121 (28) |
| Comorbidity (SCQ, 0-15) | 1.64 (1.39-1.95) | 15 (3) | 1.54 (1.21-1.96) | 121 (28) |
| Radiating pain below knee (ref: no) | 2.60 (1.66-4.08) | 0 (0) | 1.94 (1.09-3.44) | 121 (28) |
| Health-related QOL physical (SF36, 0-100) |  | 36 (8) |  | 145 (33) |
| 4. percentile, scores of >47.2-100 (ref.) | 1.00 |  | 1.00 |  |
| 3. percentile, scores of >42.1-47.2 | 2.71 (1.22-6.03) |  | 2.06 (0.75-5.68) |  |
| 2. percentile, scores of >35.6-42.1 | 3.54 (1.61-7.78) |  | 1.81 (0.65-5.06) |  |
| 1. percentile, scores of 0-35.6 | 6.57 (3.06-14.13) |  | 4.79 (1.83-12.53) |  |
| Health-related QOL mental (SF36, 0-100) |  | 36 (8) |  | 145 (33) |
| 4. percentile, scores of >59.7-100 (ref.) | 1.00 |  | 1.00 |  |
| 3. percentile, scores of >54.9-59.7 | 0.87 (0.43-1.79) |  | 1.15 (0.46-2.87) |  |
| 2. percentile, scores of >47.6-54.9 | 1.87 (0.97-3.59) |  | 1.96 (0.85-4.56) |  |
| 1. percentile, scores of 0-47.6 | 2.58 (1.35-4.94) |  | 3.56 (1.51-8.42) |  |
| Expectations of recovery within 3 months |  | 17 (4) |  | 122 (28) |
| Recovered (ref.) | 1.00 |  | 1.00 |  |
| Much better | 2.53 (1.39-4.57) |  | 1.18 (0.56-2.46) |  |
| No change or worse | 1.67 (0.84-3.33) |  | 1.45 (0.62-3.39) |  |
| CES-D indicates The Center for Epidemiologic Studies Depression Scale; CI, confidence interval; FABQ-PA, The Fear Avoidance Beliefs Questionnaire, physical activity subscale; NRS, Numeric Rating Scale; OR, odds ratio; RMDQ, The Roland Morris Disability Questionnaire; SCQ, The Self-Administered Comorbidity Questionnaire; SF-36, The Short-Form Health Survey 36-item. *Adjusted by sex, age, education level, employment status, pain duration, pain history, first healthcare provider and costs related to healthcare utilization prior to inclusion. | | | | |
